# Upfront Radiotherapy with Concurrent and Adjuvant Vismodegib Is Effective and Well-Tolerated in a Patient with Advanced, Multifocal Basal Cell Carcinoma

**DOI:** 10.1155/2018/2354146

**Published:** 2018-02-21

**Authors:** Abigail I. Franco, Gary Eastwick, Ramsay Farah, Marvin Heyboer, Mijung Lee, Paul Aridgides

**Affiliations:** ^1^St. Joseph's Hospital Health Center, 301 Prospect Avenue, Syracuse, NY 13203, USA; ^2^Upstate University Hospital, 750 E. Adams Street, Syracuse, NY 13210, USA

## Abstract

We present a case report of a male with multifocal and extensive basal cell carcinoma. Due to extremely large size and deep tumor infiltration, he was not a surgical candidate. Combined modality treatment of fractionated radiation with concurrent vismodegib was chosen. Concurrent treatment was previously reported in the* palliative* and* recurrent* setting. This is the first case of concurrent vismodegib and radiation therapy for* upfront definitive* management. The patient experienced complete response in all treated lesions.

## 1. Introduction

Basal cell carcinoma (BCC), the most common skin malignancy, has an estimated incidence of 226 cases per 100,0000 individuals yearly in the United States [1]. Surgical excision is extremely effective with <5% of patients developing local failure [2]. Radiation therapy (RT), while similarly curative for smaller tumors (95%), is more frequently used for larger tumors where size > 10 mm has been associated with lower efficacy (90%) [3]. As would be expected, even more advanced tumors are associated with higher rates of failure. In a retrospective review of 115 advanced BCCs treated with RT, where the median tumor size was 7 cm (range 3 to 32 cm), the 5-year cure rate dropped to 55%  [4]. A recent consensus from a UK panel of experts has defined advanced BCC as being BCC stage II or above (as categorized by the American Joint Committee on Cancer as a tumor larger than 2 cm with no metastases), in which current treatment modalities may be contraindicated by clinical or patient-driven factors [4].

Vismodegib is a small molecule inhibitor of smoothened homologue (SMO) and has shown clinical efficacy in the treatment of locally advanced or metastatic BCC in patients who are not candidates for or have failed prior treatment with surgery and/or radiation [5]. In the Landmark phase II trial proving such efficacy, vismodegib led to treatment response in 43% of patients with locally advanced BCC, all of whom had previously failed RT [5]. Given the low cure rate using vismodegib alone following RT, and given the correlation of tumor size with local failure following primary radiotherapy alone [4, 6], we chose to administer vismodegib* concurrently* with electron beam radiation in the primary treatment of a patient with multiple extremely large BCC tumors of the torso. Previous reports have shown concurrent therapy to be effective in either the recurrent or metastatic setting [7, 8], but concurrent therapy has not been previously reported for upfront treatment until this case.

## 2. Case Presentation

### 2.1. Presentation

**Figure 1:**
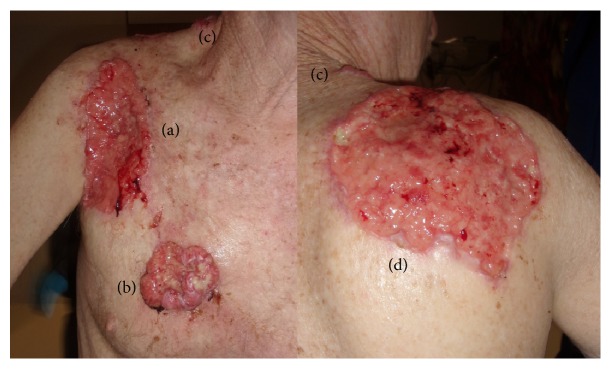
Large ulcerative lesions of the right thorax, with subcutaneous fat layer exposed, upon presentation: anterior upper ((a), size 15 × 9 cm), anterior lower ((b), 6 × 7 cm), and lower neck seen both in anterior and posterior views ((c), 1 × 1 cm) and the back ((d), 15 × 12.5 cm). One additional lesion in the left back (3.5 × 2 cm) is not shown. There is evidence of invasion to the underlying muscle (a, d) and pronounced nodularity (a, c, d).

A male mason in his fifties presented with pain, shortness of breath, easy fatigability, generalized weakness, lightheadedness, and twenty-pound weight loss. He reported bleeding over several years from multiple skin lesions of the anterior (15 × 9 cm) and posterior (15 × 12.5 cm) right shoulder, right chest (6 × 7 cm), right neck (1 × 1 cm), and left back (3.5 × 2 cm) that correlated with significant occupational ultraviolet light exposure (see Figure 1). His hemoglobin on admission was 3.1 g/dL. The origin of the patient's severe anemia and associated constitutional symptoms was consistent with chronic bleeding from multifocal skin cancer. He was resuscitated with 7 units of packed red blood cells and metastatic work-up, consisting of computed tomography of the chest abdomen and pelvis, was negative. Pathologic biopsy of two lesions revealed ulcerated BCC, usual and infiltrating types. Surgical resection was not technically feasible due the extremely large skin surface area involved with deep muscle invasion. In multidisciplinary consultation with dermatology, radiation oncology, and medical oncology, concurrent radiation and vismodegib were recommended. The patient provided informed consent for the treatment modality as well as for being the subject for this case report.

### 2.2. Treatment

Radiotherapy was delivered using mixed six- and nine-mega-electron-volt (MeV) energy electrons to 66 Gray (Gy) in 2 Gy fractions (33 fractions) prescribed to the 90% isodose line to five separate lesions on the right and left back, right neck, and right anterior chest wall. Radiation was started urgently due to severe pain and bleeding. He was planned for upfront concurrent vismodegib (150 mg) which was initiated immediately following insurance approval, 3 weeks into his radiation treatment, on fraction 16 of 33. Following treatment, the patient reported nearly complete resolution of pain and bleeding, with significant regression of all treated lesions. One lesion, on the inferior right chest wall, had residual nodularity and was boosted to a total of 76 Gy with four additional 2.5 Gy fractions three weeks after finishing his initial radiation. During his course of radiation, the patient was treated with antibiotic therapy for secondary impetigo.

Vismodegib was continued adjuvantly for three months, until discontinuation due to development of grade 3 diarrhea. His diarrhea was likely multifactorial since it resolved with discontinuation of vismodegib and successful treatment of* Clostridium difficile* colitis. Vismodegib was restarted after a 2-month break, initially on an every-other-day schedule for 1 month, and then increasing to once daily for 2 months, until GI upset recurred and the medication was discontinued.

**Figure 2:**
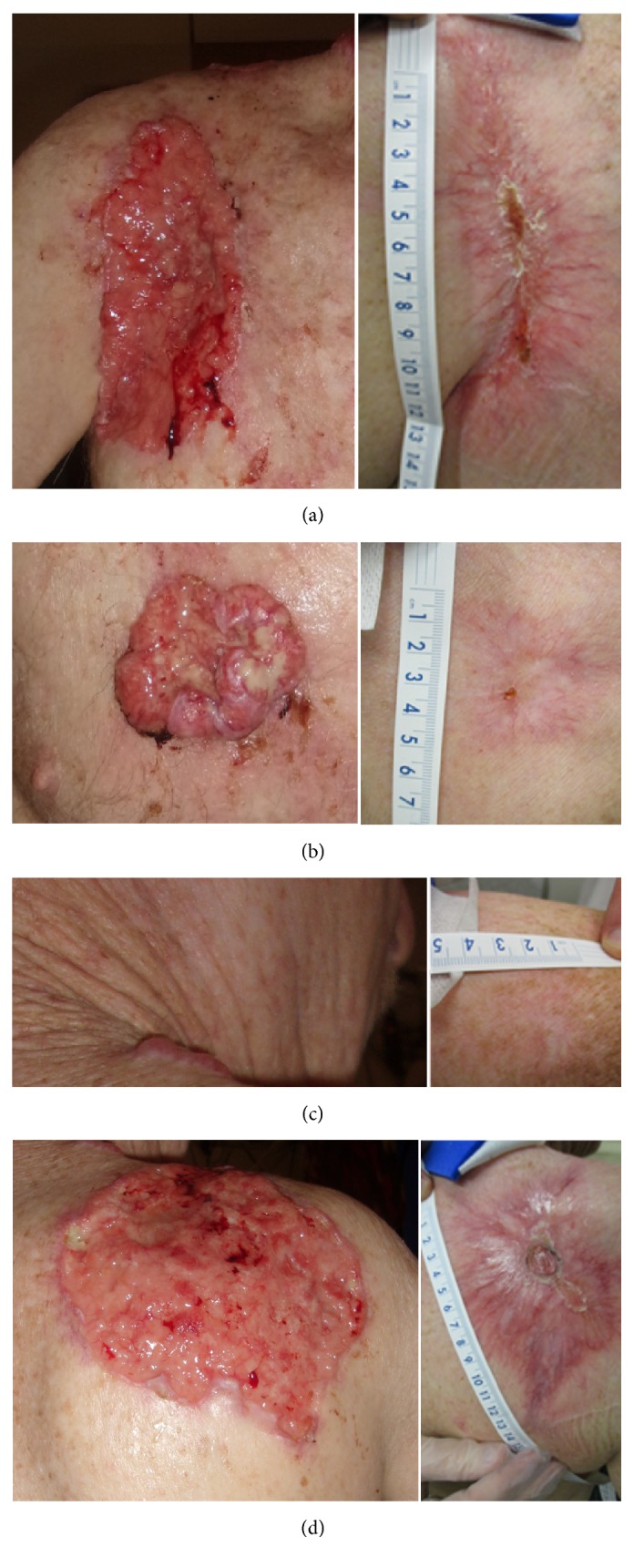
Lesions of the right thorax before (left) and six months after (right) radiation showing excellent response to radiation (66–76 Gy) with concurrent and adjuvant vismodegib: (anterior upper (a); anterior lower (b); lower neck (c); and back (d)). All lesions completely resolved with complete skin closure, including an additional left back lesion (not shown).

By 6 months following radiation treatment, all lesions showed complete response with near-complete skin closure (Figure 2). The patient reported no residual pain but had moderate residual impairment in right shoulder abduction secondary to fibrosis of the skin/soft tissues and/or initial muscle involvement. Healing was achieved with the careful attention of a dedicated wound care service through the university hospital wound care center. The patient was followed for a period of 10 months. The largest ulcers on the right chest (a) and right upper back (d) were measured at 15 cm × 9 cm × 0.3 cm and 15 cm × 12.5 cm × 0.2 cm initially. They were treated with serial surgical debridement in the outpatient setting initially weekly and then biweekly. In addition, a moisture retentive dressing with antimicrobial/antifungal properties was applied every other day. Finally, a placenta derived tissue substitute application was used at 6 months. This resulted in objectively decreasing surface area to the point of complete healing. Following healing, the patient was to practice good skin care including application of moisturizing lotion twice daily.

## 3. Discussion

Radiation therapy (RT) is an extremely effective modality for BCC; however efficacy decreases with advanced lesions. Larger tumor size correlates with lower frequency of achieving complete remission (complete disappearance of the lesion upon first follow-up), showing only 70% complete remission for extensive BCCs [4]. Additionally, whereas typical BCC lesions treated with RT show a 5-year cure rate of 84–96%, this rate seems to decrease significantly with very large tumors to as low as 55%[4]. More aggressive types of BCC (including infiltrative BCC, as seen in our patient) are likely a contributing factor to the worse 5-year cure rates seen in advanced BCC [4].

Due to the aggressive nature of the BCC in our patient (size and histology) and the lower cure rates in such cases, we chose combined modality treatment using definitive radiotherapy with concurrent and adjuvant vismodegib. Vismodegib's efficacy following RT in locally advanced BCC is reported to be only 43% [5]. Our hope with this patient's treatment plan was that combining vismodegib and RT* concurrently* might prove more efficacious. We performed several PubMed searches to examine any evidence for concurrent vismodegib and RT and found one report (two patients) by Pollom et al. which showed efficacy for concurrent external beam radiation and vismodegib in the recurrent setting [7, 8]. Our case differs in its multifocal nature, higher burden of disease, and upfront (rather than recurrent) setting. To our knowledge, our case represents the first report of upfront definitive treatment for advanced BCC using concurrent RT and vismodegib. Even with the patient's extensive tumor volume, occupying an estimated 10–15% of the thorax skin surface area, complete remission and wound closure were achieved for all lesions.

Vismodegib is currently approved for patients with metastatic or locally advanced BCC who are not candidates for or who have had disease recurrence after surgery and/or radiation therapy [5]. Little is known about the interaction of radiation with vismodegib. Duration-limiting side effects have been well-documented with the use of vismodegib [5]. Combining treatment with radiation therapy may prove helpful since long-term treatment with vismodegib may not be realistic. This patient tolerated a total of six months of vismodegib with a two-month vismodegib-free vacation in the middle and using every-other-day dosing when restarting the treatment. The patient discontinued vismodegib twice due to GI upset. However, it is notable that the wounds showed significant improvement after vismodegib was restarted. The hedgehog pathway has been implicated in the wound healing process and it has previously been suggested that vismodegib may have potential to exacerbate radiation's side effect of slow wound healing [8, 9]. However this is contrary to our case, where the patient's extensive wounds healed entirely after combined vismodegib and radiation therapy. It is noted that this patient saw wound care first weekly and then twice monthly, which certainly contributed to his successful wound healing.

There is currently an open phase II trial investigating the safety and tolerability of combined vismodegib and radiotherapy in the setting of locally advanced head and neck basal cell carcinoma with neck nodal involvement. Our case report demonstrates the tolerability and efficacy of definitive doses of radiation to large areas of skin with concurrent vismodegib in treating multifocal large inoperable BCCs. It should be noted that the patient did not undergo rebiopsy or reimaging studies following his treatment; however, the patient was followed clinically until disappearance of all visible tumor and complete skin healing. This is our standard surveillance regimen for primary radiation, where we reserve biopsy for cases of incomplete response
